# Supplementary material for: Extracorporeal Shock Wave Therapy Salvages Critical Limb Ischemia in B6 Mice through Upregulating Cell Proliferation Signaling and Angiogenesis
Source: Biomedicines. 2022 Jan 6;10(1):117. doi: 10.3390/biomedicines10010117 (PMC8773589; doi:10.3390/biomedicines10010117)
Supplement: Supplementary file 1 [file biomedicines-10-00117-s001.zip › biomedicines-1493721-supplementary.pdf]

Supplementary Materials

Extracorporeal Shock Wave Therapy Salvaged the Critical Limb Ischemia in B6 Mice through Upregulating the Cell Proliferation Signaling and Angiogenesis

Pei-Hsun Sung [1,2,3,†], Tsung-Cheng Yin [4,†], Han-Tan Chai [1], John Y. Chiang [5,6], Chih-Hung Chen [7], Chi-Ruei Huang [1,2] and Hon-Kan Yip [1,2,8,*]

1 Division of Cardiology, Department of Internal Medicine, Kaohsiung Chang Gung Memorial Hospital, Chang Gung University College of Medicine, Kaohsiung, Taiwan. e12281@cgmh.org.tw (P.-H.S.); chaiht@mail.cgmh.org.tw (H.-T.C.); starchmay33@gmail.com (C.-R.H.)
2 Center for Shockwave Medicine and Tissue Engineering, Kaohsiung Chang Gung Memorial Hospital, Kaohsiung, Taiwan.
3 Institute for Translational Research in Biomedicine, Kaohsiung Chang Gung Memorial Hospital, Kaohsiung, Taiwan.
4 Department of Orthopedics, Kaohsiung Chang Gung Memorial Hospital and Chang Gung University College of Medicine, Kaohsiung, Taiwan. tsongchenyin@gmail.com.
5 Department of Computer Science and Engineering, National Sun Yat-Sen University, Kaohsiung, Taiwan. chiang@cse.nsysu.edu.tw
6 Department of Healthcare Administration and Medical Informatics, Kaohsiung Medical University, Kaohsiung, Taiwan.
7 Divisions of General Medicine, Kaohsiung Chang Gung Memorial Hospital, Chang Gung University College of Medicine, Kaohsiung, Taiwan. starchmay33@gmail.com
8 Department of Medical Research, China Medical University Hospital, China Medical University, Taichung, Taiwan.
† These authors contributed equally to this work.
* Correspondence: han.gung@msa.hinet.net; Tel: +886-7-7317123, Fax: +886-7-7322402

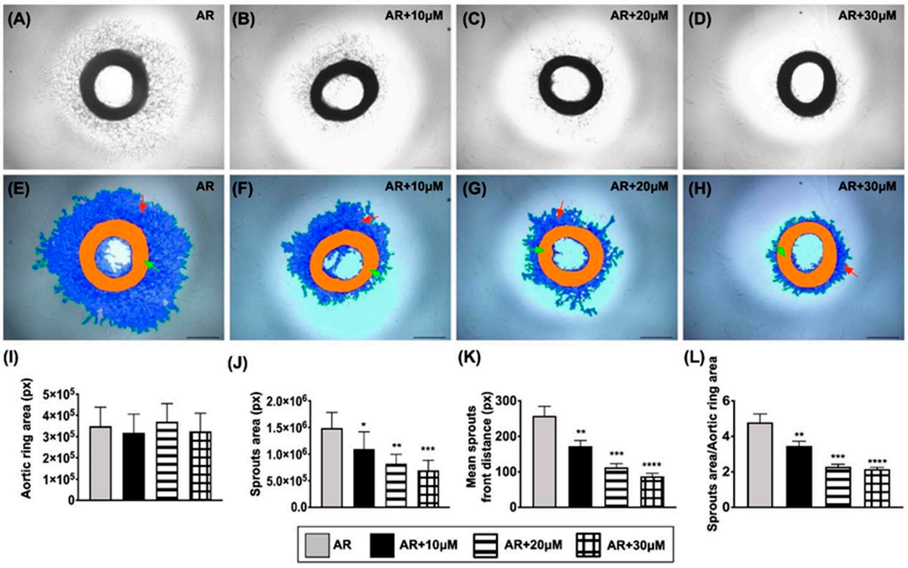

Figure S1. Ex vivo study of rat aortic ring assay for assessing the impact of Avastin on suppressing angiogenesis (**A**–**H**) Illustrating the results of 5-day exo vivo culture of aortic ring (AR) for determining the angiogenesis in groups 1 (**A**,**B**), 2 (**C**,**D**), 3 (**E**,**F**) and 4 (**G**,**H**), respectively. Scale bars in right lower corner represent 200 μm. The orange color (green arrows) indicated AR area. The blue color (red arrows) indicated sprout area. (I) Analytical result of AR area, $p > 0.5$. (**J**) Analytical result of sprout area, * for p<0.05, ** for p<0.01, *** for p<0.001. (**K**) Analytical result of Mean sprouts front distance, ** for p<0.01, *** for p<0.001, **** for p<0.0001. (**L**) Analytical result of the ratio of sprout area to AR area, ** for p<0.01, *** for p<0.001, **** for p<0.0001. Wimasis image analysis (Wimasis GmbH: Limited Liability Company) was utilized for quantitative analysis (*n* = 4 for each group).

group 1 = Aortic ring (AR) (i.e., sham-control); group 2 = AR + Avastin (10 μM); group 3 = AR + Avastin (20 μM); group 4 = AR + Avastin (30 μM).

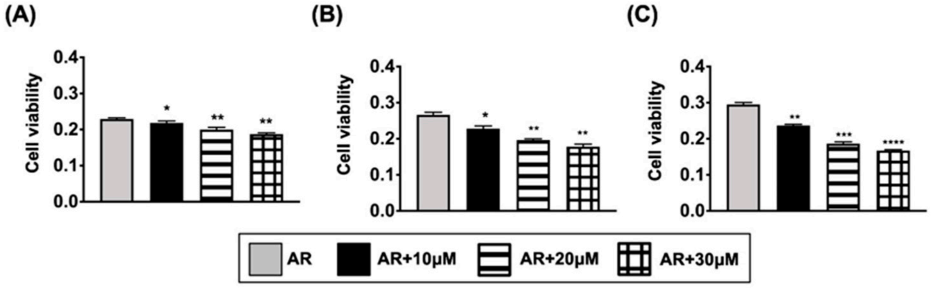

Figure S2. MTT assay for determining the cell viability (**A**–**C**) By 24h (**A**), 48h (**B**) and 72h (**C**), cell culturing, the cell viability was significantly progressively reduced from groups 1 to 4, * for $p < 0.05$, ** for $p<0.01$, *** for $p<0.001$, **** for $p < 0.0001$ ($n = 4$ for each group). MTT = [3-(4,5-Dimethylthiazol-2-yl)-2,5-diphenyltetrazolium bromide]. group 1 = Aortic ring (AR) (i.e., sham-control); group 2 = AR + Avastin (10 μM); group 3 = AR + Avastin (20 uM); group 4 = AR + Avastin (30 μM).
